# Marathon Running Increases Synthesis and Decreases Catabolism of Joint Cartilage Type II Collagen Accompanied by High-Energy Demands and an Inflamatory Reaction

**DOI:** 10.3389/fphys.2021.722718

**Published:** 2021-10-11

**Authors:** José A. Hernández-Hermoso, Lexa Nescolarde, Emma Roca, Elena Revuelta-López, Jordi Ara, Antoni Bayes-Genis

**Affiliations:** ^1^Department of Orthopedic Surgery and Traumatology, Hospital Universitari Germans Trias i Pujol, Barcelona, Spain; ^2^Department of Surgery, Faculty of Medicine, Universitat Autònoma Barcelona, Bellaterra, Spain; ^3^Department of Electronic Engineering, Universitat Politècnica de Catalunya, Barcelona, Spain; ^4^Summit 2014 SL, Barcelona, Spain; ^5^Research Program, Fundació Institut d'Investigació en Ciències de la Salut Germans Trias i Pujol, Barcelona, Spain; ^6^Departament of Medicine, Faculty of Medicine, Universitat Autònoma de Barcelona, Barcelona, Spain; ^7^Department of Nephrology, Hospital Universitari Germans Trias i Pujol, Barcelona, Spain; ^8^Department of Cardiology, Hospital Universitari Germans Trias i Pujol, Barcelona, Spain

**Keywords:** biomarker, marathon runners, articular cartilage, PIINP, C2C, hyaluronic acid

## Abstract

**Objective:** To determine the effect of marathon running on serum levels of inflammatory, high energy, and cartilage matrix biomarkers and to ascertain whether these biomarkers levels correlate.

**Design:** Blood samples from 17 Caucasian male recreational athletes at the Barcelona Marathon 2017 were collected at the baseline, immediately and 48 h post-race. Serum C reactive protein (CRP), creatin kinase (CK), and lactate dehydrogenase (LDH) were determined using an AU-5800 chemistry analyser. Serum levels of hyaluronan (HA), cartilage oligomeric matrix protein (COMP), aggrecan chondroitin sulphate 846 (CS846), glycoprotein YKL-40, human procollagen II N-terminal propeptide (PIINP), human type IIA collagen N-propeptide (PIIANP), and collagen type II cleavage (C2C) were measured by sandwich enzyme-linked immune-sorbent assay (ELISA).

**Results:** Medians CK and sLDH levels increased (three-fold, two-fold) post-race [429 (332) U/L, 323 (69) U/L] (*p* < 0.0001; *p* < 0.0001) and (six-fold, 1.2-fold) 48 h post-race [658 (1,073) U/L, 218 (45) U/L] (*p* < 0.0001; *p* < 0.0001). Medians CRP increased (ten-fold) after 48 h post-race [6.8 (4.1) mg/L] (*p* < 0.0001). Mean sHA levels increased (four-fold) post-race (89.54 ± 53.14 ng/ml) (*p* < 0.0001). Means PIINP (9.05 ± 2.15 ng/ml) levels increased post-race (10.82 ± 3.44 ng/ml) (*p* = 0.053) and 48 h post-race (11.00 ± 2.96 ng/ml) (*p* = 0.001). Mean sC2C levels (220.83 ± 39.50 ng/ml) decreased post-race (188.67 ± 38.52 ng/ml) (*p* = 0.002). In contrast, means COMP, sCS846, sPIIANP, and median sYKL-40 were relatively stable. We found a positive association between sCK levels with sLDH pre-race (*r* = 0.758, *p* < 0.0001), post-race (*r* = 0.623, *p* = 0.008) and 48-h post-race (*r* = 0.842, *p* < 0.0001); sHA with sCRP post-race vs. 48 h post-race (*r* = 0.563, *p* = 0.019) and sPIINP with sCK pre-race vs. 48-h post-race (*r* = 0.499, *p* = 0.044) and with sLDH 48-h pre-race vs. post-race (*r* = 0.610, *p* = 0.009) and a negative correlation of sPIIANP with sCRP 48-h post-race (*r* = −0.570, *p* = 0.017).

**Conclusion:** Marathon running is an exercise with high-energy demands (sCK and sLDH increase) that provokes a high and durable general inflammatory reaction (sCRP increase) and an immediately post-marathon mechanism to protect inflammation and cartilage (sHA increase). Accompanied by an increase in type II collagen cartilage fibrils synthesis (sPIINP increase) and a decrease in its catabolism (sC2C decrease), without changes in non-collagenous cartilage metabolism (sCOMP, sC846, and sYKL-40). Metabolic changes on sPIINP and sHA synthesis may be related to energy consumption (sCK, sLDH) and the inflammatory reaction (sCRP) produced.

## Introduction

Despite the substantial increase in marathon runners, still, it is not known what is a physiological or expected acute inflammatory or cartilage metabolic response to an increase in physical activity (Cattano et al., 2017b; Driban et al., 2017).

Long-distance running imposes high levels of stress on the musculoskeletal system, causing changes in cartilage volume (Boocock et al., 2009; Doré et al., 2013) and numerous cellular and molecular changes (Cattano et al., 2017b). The intensive training and the marathon itself induce inflammation and muscle fibre necrosis (Neidhart et al., 2000). Blood-circulating biomarkers used to monitor inflammation and joint homeostasis change after joint loading with different types of activity level or sports, and offer a method to analyse cartilage metabolic response to physical activity, although data available give contrasting results (Cattano et al., 2017b). The discrepancy of these results may be attributable to differences in the study methods and to many confounding factors such as ethnicity (Jordan et al., 2003), gender (Jordan et al., 2003; Niehoff et al., 2010), body weight (Matt Denning et al., 2015), activity time or level (Cattano et al., 2017a), loading protocols (Niehoff et al., 2010), vibration (Cattano et al., 2017b), food intake (Gordon et al., 2008), diurnal variation (Andersson et al., 2006), and osteoarthritis (Chu et al., 2018); further, biomarkers may adapt to training over time (Cattano et al., 2017b). To date, the correlation between inflammatory, energy enzymes, and collagenous and non-collagenous cartilage markers after a marathon run is unknown.

Inflammatory and joint homeostasis (anabolic and catabolic) biomarkers can be informative about the overall physiological state of a patient but are less informative for determining if a specific joint of a patient is failing to adapt to a given loading stimulus (Cattano et al., 2017b). Since metabolic changes of joint tissues begin long before the onset of structural alterations, biomarker responses to physical activity may serve as indicators of tolerance (or intolerance) to physical activity and could potentially be utilised to help in prediction of cartilage loss from post-walking (Erhart-Hledik et al., 2012) or in developing new therapeutic alternatives to maintain cartilage thickness as vibration training (Liphardt et al., 2009).

Inflammatory biomarkers as C-reactive protein (CRP), an acute-phase inflammatory protein, and high-energy enzyme biomarkers as creatin kinase (CK), a high-energy phosphate enzyme, reportedly have a dose-dependent behaviour with a transient increase after a 24-h marathon and ultra-marathon (Kim et al., 2009). Lactate dehydrogenase (LDH) is a key glycolytic enzyme that increases with increased training loads (Radzimiński et al., 2020).

Mechanosensitive non-collagenous joint homeostasis biomarkers as the hyaluronic acid (HA) have shown that their serum level decreases within 1 h of walking (Pruksakorn et al., 2013) on healthy individuals. COMP seems to have a temporary dose-dependent increase in response to physical activity with a gradual post-activity return to normal (Mündermann et al., 2005; Erhart-Hledik et al., 2012; Celik et al., 2013; Pruksakorn et al., 2013; Matt Denning et al., 2015; Liphardt et al., 2018). CS846, an aggrecan synthesis marker, after a 30-min walk, correlates with cartilage thickening of the lateral femur at 5-year follow-up (Chu et al., 2018). Human chitinase-3-like-1 protein, YKL-40 synthesis is related to alteration of cartilage in osteoarthritis (Di Rosa et al., 2013) and to altered joint mechanical conditions after anterior cruciate transection in dogs (Lorenz et al., 2005).

Mechanosensitive cartilage collagen biomarkers as the Procollagen type II N terminal pro-peptide (PIINP) is a precursor of type II collagen, a major structural protein of cartilage that exists in two splice variants termed PIIANP and PIIBNP; the concentration of these peptides assesses biosynthetic activity (Munk et al., 2014). The cleavage of type II collagen by collagenases yields fragments, such as the C2C epitope, that correlate with cartilage degeneration (King et al., 2004). Well-rounded exercise may have beneficial effects on type II collagen metabolism (Azukizawa et al., 2019); however, the behaviour of metabolites, such as PIINP, PIIANP, and C2C, after load or sports has not been investigated.

The aim of the study was to extend knowledge about the response of serum levels of inflammatory proteins, high-energy enzymes, and synthesis and degradation of non-collagenous and type II collagenous cartilage biomarkers pre- and post-marathon running in healthy non-elite athletes and to ascertain whether they correlate. We hypothesised marathon running would increase the metabolism of CRP (inflammatory), CK, LDH (high-energy), COMP, CS846, YKL40 (non-collagenous), PIINP, and PIIANP (type II collagen) biomarkers and decrease HA (non-collagenous) and C2C (cartilage degradation) biomarkers. We further hypothesised that inflammatory and energy metabolic biomarkers increase may be related with non-collagenous and type II collagenous cartilage biomarkers metabolic changes.

## Materials and Methods

We examined the dynamics of a panel of inflammatory, high-energy, and cartilage biomarkers in non-elite athletes completing the Barcelona Marathon in 2017. These new data were recorded as part of the SUMMIT Project, whose main goal is analysing health risk in the population engaged in high-intensity physical activities (Maqueda et al., 2017; Roca et al., 2017, 2019; Nescolarde et al., 2020). The procedures followed were in accordance with the ethical standards of the responsible committee on human experimentation (institutional and national) and with the Helsinki Declaration of 1975, as revised in 2000. The local ethics committee approved the study (approval No. PI-17-037), and all the participants provided written informed consent.

A total of 35 runners, 29 male and 6 females, responded to a call for volunteers. The participants were excluded if time race was slower than the third pacemaker (3:15 h) in Barcelona Marathon 2016 or if they had lower limb joints symptoms 6 months previous to marathon, any history of previous lower limb injury or surgery, or if they have taken any medication, including nonsteroidal anti-inflammatory drugs, in the previous month and during the 48-h post-marathon. We recommended no intense physical exercise for the 48-h period before and after the marathon race. A checklist was used to supervise medication, food intake, and exercise constraints, which were compiled by all the runners. The study sample included 17 healthy Caucasian male recreational athletes (mean age 41 ± 4 years old; 77.4 ± 7.1 kg; 1.80 ± 0.1 m; 24. ± 2.1 kg/m^2^) that ran 2.7 ± 1.6 h/of intensive training per week and had a running history of 8.2 ± 5.1 years, on average, prior to the race.

The Barcelona Marathon 2017 (42.195 km on asphalt at the sea level) began at 8:30 at a temperature of 11–14°C and 68–79% humidity. During the race, the runners maintained adequate levels of hydration by eating and drinking at aid stations every 5 km, according to pre-established guidelines, with such sustenance as mineral water, sports drinks, fruits, and nuts. The first liquid intake was programmed at 60 min of race: 400 ml for lighter/slower runners and 800 ml for heavier/faster runners, drinking 100–150 ml every 15–20 min. Commercialised beverages were provided for runners with an average of 480 mg/l for Na^+^, 85 mg/l for K^+^, and 45 mg/l for Mg^2+^. Race time of our runners was 03:29:36 ± 00:17:06. Race time recorded of each participant was the official time from the race organisation (Zurich Barcelona Marathon).

Blood samples were collected by a certified phlebotomist from an antecubital vein immediately after arrival to the laboratory. The area was cleaned with isopropyl alcohol, then the needle was inserted, and blood was drawn into an 8.5 ml, 16 × 100-mm Vacutainer® BD® tube with SST^TM^ II Advance gel (reference 5T02.367953) at the baseline 24–48 h before the race, immediately post-race and 48 h post-race. Non-fasting blood samples were collected at the same time of the day (from 11:30 h to 13 h am). Before the pre and 48-h post-race blood sample draw, the subjects were recommended to have breakfast, after overnight fasting, with sports drinks, fruits, and nuts from 9 to 10 am. The blood samples were centrifuged at 3,000 rpm at 4°C for 10 min in a bench-top centrifuge. The supernatant serum was aliquoted and stored in dry ice until samples were frozen at −80°C in sealed Eppendorf tubes to avoid evaporation until analysis. The laboratory analysis was done in the IGTP laboratories that is adhered to standards in biosecurity and safety procedures.

### Measurements of Inflammatory and High-Energy Enzymes Biomarkers

Serum C reactive protein (CRP), CK, and LDH were determined using an AU-5800 Chemistry Analyzer (Beckman Coulter Inc, CA, United States) before freezing the sample.

### Measurements of Non-collagenous and Type II Collagenous Cartilage Biomarkers

The quantitative detection of serum cartilage biomarkers was carried out by sandwich enzyme-linked immune-sorbent assay technology (ELISA). Biomarker analysis was performed according to the manuals of the manufacturer. Singlet data analysis was carried out using duplicate standards; the acceptance criteria were that the coefficient of variance of mean calculated concentrations between single result, and duplicate mean should be <15%. Duplicate standards were loaded at the beginning and the end of the plate. First, the samples, standards, and controls were loaded on an inert plate and then with a multi-channel pippete, in less than a minute; the samples were passed to the ELISA plate. In addition, all analyses were carried out by the same technical person and on ELISA plates from the same batch of each biomarker to reduce intra- and inter- assay variation, respectively. All samples were analysed in the first or second thaw-defrost cycle. Biomarker analysis staff were blinded to any data related to participating subjects.

Measurements of hyaluronic acid (HA) were performed with the HA ELISA Kit (Abbexa Ltd., Cambridge Science Park, Cambridge, UK; code No. abx257152, batch No. E1904223U). Serum aliquots were diluted 1:4 in a dilution buffer provided by the manufacturer before incubation. The standard range and sensitivity are between 1.56–100 and <0.938 ng/ml, respectively. Intra and inter-assay precisions were <8 and <10%, respectively.

Human COMP was analysed by COMP ELISA Kit (Abbexa Ltd., Cambridge Science Park, Cambridge, UK; code No. abx151136, batch No. E1904400E). Serum aliquots were diluted 1:20 in PBS (pH 7.0–7.2). Assay range is between 3.12 and 200 ng/ml. Sensitivity in this assay is <1.21 ng/ml. Intra and inter-assay precisions were <10 and 12%, respectively.

Aggrecan chondroitin sulphate 846 (CS846) was measured by the CS846 epitope ELISA (IBEX Pharmaceuticals Inc., Quebec, Canada; code No. #60-1004; batch No. #2019-01). This assay is a competitive enzyme immunoassay. Serum samples were diluted 1:5 in a dilution buffer provided by the manufacturer. The standard range is 20–1,000 ng/ml.

Human chitinase-3-like protein 1 (CHI3L1) levels were analysed by the Glycoprotein YKL-40 ELISA kit (Blue Gene, Shanghai, China; code No. E01C0072, batch No. BG190510). Samples were loaded undiluted. The standard range and sensitivity are 2.5–50 and 1 ng/ml, respectively.

Measurements of human procollagen II N-terminal propeptide (PIINP) were performed with the PIINP ELISA Kit (Abbexa Ltd., Cambridge Science Park, Cambridge, UK; code No. abx152741, batch No. E1904136K). Samples were diluted 1:10 in PBS (pH 7–7.2). The standard range is 93.75–6,000 pg/ml, and the sensitivity is <37.55 pg/ml. Intra and inter-assay precisions were <10 and 12%, respectively.

Human type IIA collagen N-propeptide (PIIANP) levels were analysed by Human PIIANP Kit (Creative Diagnostics®, New York, EEUU; code No. DEIA7442, batch No. 3175077). Samples were diluted 1:2 in an assay buffer. The sensitivity is 39 ng/ml. Intra and inter-assay variations were 3.37–6.60 and 4.78–7.77%, respectively.

Collagen Type II Cleavage (C2C) was analysed by a competitive assay, C2C ELISA Kit (IBEX Pharmaceuticals Inc., Quebec, Canada; code No. #60-1001-001; batch No. #2019-02). Samples were diluted 1:2 in a dilution buffer provided by the manufacturer. The calibration range is 10–1,000 ng/ml.

### Statistical Analysis

The normality of distribution of the variables was checked by the Shapiro-Wilk test and homogeneity of variances by Levene's test. The variables normally distributed are shown as mean ± SD, 95% confidence interval for mean (lower and upper bound), while those non-normally distributed data are shown as median, interquartile range (IQR) (minimum–maximum). The serum biomarker levels were normally distributed, except for CK, LDH, CRP, and YKL-40.

Repeated measures ANOVA test was used to evaluate the effect of inflammation proteins, high-energy enzymes, non-collagenous and collagenous cartilage proteins biomarkers measured 24-h pre-race (1), immediately post-race (2), and 48-h post-race (3), with multiple comparison test by Bonferroni. The Friedman test was used for the repeated measure of data non-normally distributed with the Wilcoxon test.

In addition, we made Spearman Rho correlations between immediately post-race (2) with respect to 24-h pre-race (1); 48-h post-race (3) with respect to 24-h pre-race (1); and 48-h post-race (3) with respect to immediately post-race (2) for all the biomarkers and time points and presented the significant ones for the biomarkers that showed a statistically significant change.

Data were analysed using SPSS for windows version 26.0 statistical program (SPSS, Inc., Chicago, IL). The statistical significance was set at *p* < 0.05.

## Results

The 17 study participants completed all data collection time points.

### Inflammatory and High-Energy Enzymes Biomarkers

Pre-race median sCK and sLDH levels increased immediately post-race, up to three-fold and two-fold increase, respectively (*p* < 0.0001) [10 (59%) runners over three-fold and 12 (71%) over two-fold, respectively]. The level of sCK remained elevated 48-h post-race compared to immediately post (two-fold increase) (*p* < 0.0001) and pre-race (four-fold increase) (*p* < 0.0001) [8 (47%) runners over four-fold]. In contrast, sLDH median levels decreased 48 h post-race relative to immediately post-race (*p* < 0.0001) but remained elevated with respect to pre-race concentration (1.2-fold increase) (*p* = 0.003) [14 (82%) runners over one-fold].

**Figure 1:**
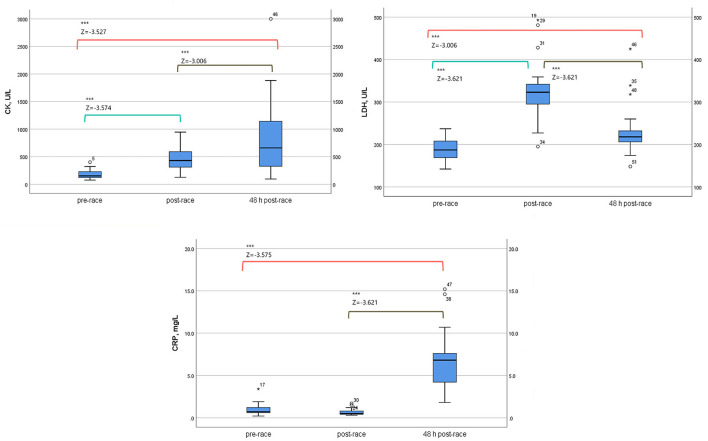
Serum energy enzymes and inflammation biomarkers levels of 17 non-elite marathon runners, pre, immediately-post, and 48-h-post marathon. CK, creatine kinase; LDH, lactate dehydrogenase; CRP, C-reactive protein. Boxplot (median, lower fence, and upper fence). Results of Wilcoxon test [Z-statistic based on negative rank and statistical significance, *p* < 0.0001 (***)].

The median sCRP increased up to ten-fold 48 h post-race compared to pre-race (*p* < 0.0001) [6 (35%) runners over ten-fold] and immediately post (*p* < 0.0001). Data is shown in Table 1 and Figure 1.

**Table 1:** Serum energy enzymes and inflammation biomarkers levels of 17 non-elite marathon runners pre- and post-marathon.

**Parameters**	**24 h pre-race (1)**	**Immediately post-race (2)**	**48 h post-race (3)**	**Friedman *p* value^a^**	**Z**	**Z**	**Z**	**Fold-change (2–1)**	**Fold-change (3-1)**
					***p* value (2–1)^b^**	***p* value (3–1)^b^**	***p* value (3–2)^b^**		
**CK (U/L)**									
Median (IQR)	152 (134)	429 (332)	658 (1073)	23.412	−3.574	−3.527	−3.006	~3	~4
(Min–Max)	(76–400)	(125–946)	(95–2999)	0.000	0.000	0.000	0.000		
**LDH (U/L)**									
Median (IQR)	187 (44)	323 (69)	218 (45)	29.059	−3.621	−3.006	−3.621	~2	~1
(Min–Max)	(142–237)	(195–493)	(148–425)	0.000	0.000	0.003	0.000		
**CRP (mg/L)**									
Median (IQR)	0.7 (0.6)	0.5 (0.6)	6.8 (4.1)	24.364	−1.851	−3.575	−3.621	~(–)1	~10
(Min–Max)	(0.2–3.4)	(0.3–1.7)	(1.8–15.2)	0.000	0.064	0.000	0.000		

a *Friedman test*;

b *Wilcoxon test*.

*CK, Creatine kinase; LDH, Lactate dehydrogenase; CRP, C-reactive protein*.

### Non-collagenous Cartilage Biomarkers

Mean sHA levels increased immediately after post-race compared with pre-race (four-fold increase) (*p* < 0.0001) [10 (59%) runners over four-fold] and returned to pre-race levels 48-h post-race.

**Figure 2:**
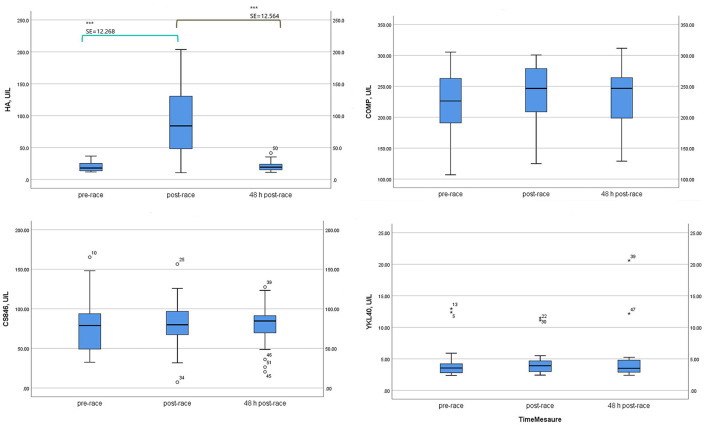
Serum non-collagenous cartilage biomarker levels of 17 non-elite runners, pre, immediately-post, and 48 h-post marathon. HA, hyaluronic acid; COMP, cartilage oligomeric matrix protein; CS846, aggrecan chondroitin sulphate 846; YKL40, Chitinase 3-like protein 1 Glycoprotein YKL-40. Boxplot (median, lower fence, and upper fence). Bonferroni pairwise comparison test (standard error, SE, and statistical significance) for HA, COMP, and CS846. Wilcoxon test (Z statistic based on negative rank and statistical significance) for YKL40. *p* < 0.0001 (***).

In contrast, sCOMP, sCS846 means, and sYKL-40 median levels were relatively stable immediately post and 48-h post-race with respect to pre-race, although mean sCOMP showed a tendency to increase (n.s.) and mean sCS846 to decrease (n.s.), immediately and 48-h post-race (Table 2; Figure 2).

**Table 2:** Serum non-collagenous cartilage biomarkers levels of 17 non-elite marathon runners pre- and post-marathon.

**Paramaters**	**24 h**	**Immediately**	**48 h**	***F***	**SE**	**SE**	**SE**	**Fold-change**	**Fold-change**
	**Pre-race**	**Post-race**	**Post-race**	***p* value^c^**	***p* value**	***p* value**	***p* value**	**(2–1)**	**(3–1)**
	**(1)**	**(2)**	**(3)**		**(1–2)^d^**	**(1–3)^d^**	**(2–3)^d^**		
**HA (ng/ml)**									
Mean ± SD	20.34 ± 7.88	89.54 ± 53.14	20.99 ± 8.04	15.002	12.268	2.377	12.564	~4	~1
95% CI									
Lowerbound	16.29	62.22	16.86	0.000	0.000	1.000	0.000		
Upperbound	24.4	116.86	25.13						
**COMP (ng/ml)**									
Mean ± SD	222.50 ± 56.86	233.43 ± 54.95	231.65 ± 49.83	0.879	8.665	12.672	13.051	~1	~1
95% CI									
Lowerbound	192.8	205.17	206.04	0.435	0.622	1.000	1.000		
Upperbound	251.27	261.68	257.27						
**CS846 (ng/ml)**									
Mean ± SD	80.25 ± 37.29	78.76 ± 34.98	78.41 ± 30.55	0.019	7.784	9.771	6.067	~(–)1	~(–)1
95% CI									
Lowerbound	61.08	60.77	62.7	0.981	1.000	1.000	1.000		
Upperbound	99.42	96.74	94.12						
**Parameters**	**24 h**	**Immediately**	**48 h**	**Friedman**	**Z**	**Z**	**Z**		
	**Pre-race**	**Post-race**	**Post-race**	***p*** **value^a^**	***p*** **value**	***p*** **value**	***p*** **value**		
	**(1)**	**(2)**	**(3)**		**(2–1)^b^**	**(3–1)^b^**	**(3–2)^b^**		
**YKL40 (pg/ml)**									
Median (IQR)	3.54 (1.47)	3.90 (2.02)	3.49 (2.30)	0.925	−0.308	−0.497	−0.207	~1	~(–)1
(Min–Max)	(2.37–12.95)	(2.42–11.50)	(2.39–20.59)	0.63	0.758	0.619	0.836		

a *Friedman test*;

b *Wilcoxon test*;

c *Repeated measures ANOVA test*;

d *Bonferroni test*.

*HA, hyaluronic acid; COMP, cartilage oligomeric matrix protein; CS846, aggrecan chondroitin sulphate 846; YKL40, Chitinase 3-like protein 1 glycoprotein YKL-40*.

### Type II Collagenous Cartilage Biomarkers

Mean sPIINP levels increased immediately (*p* = 0.053) [13 (76%) runners over one-fold] and 48-h post-race (*p* = 0.001) relative to pre-race [17 (100%) runners over one-fold].

Mean levels of sPIIANP showed a trend to decrease immediately post-race relative to pre-race, and the trend was still present 48-h post-race (*p* = 0.154).

**Figure 3:**
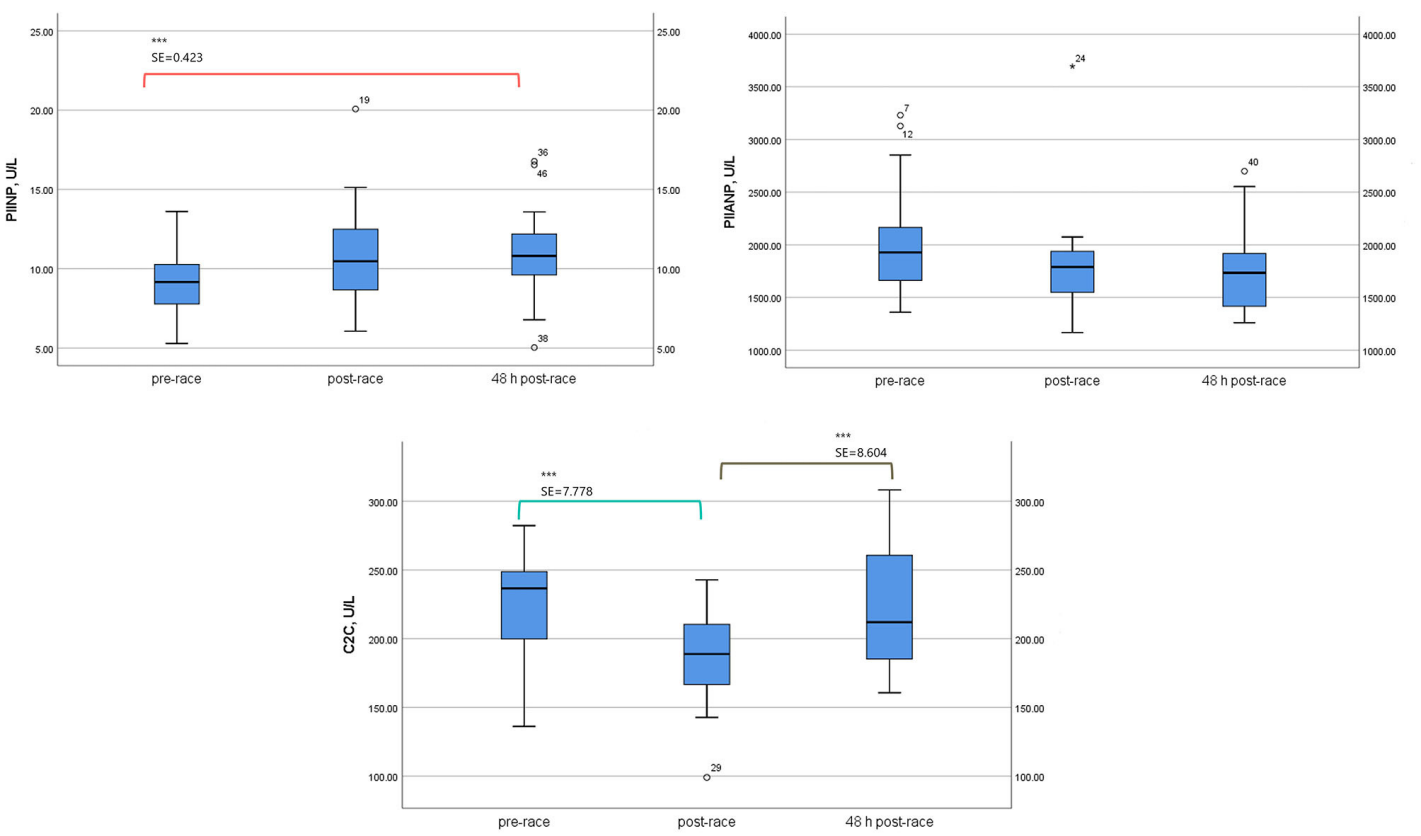
Serum type II collagenous cartilage biomarker levels of 17 non-elite runners, pre, immediately post, and 48 h-post marathon. PIIANP, Procollagen type IIA N terminal pro-peptide; C2C, cleavage type II collagen; PIINP, procollagen type II N-terminal pro-peptide. Boxplot [(median, lower fence, and upper fence). Bonferroni pairwise comparison test (standard error, SE, and statistical significance *p* < 0.01 (***)].

Mean-circulating levels sC2C was lower immediately post-race compared to pre-race (*p* = 0.002) [6 (35%) runners over one-fold] and returned to the pre-race level 48-h post-race (Table 3; Figure 3).

**Table 3:** Serum type II collagenous cartilage biomarkers levels of 17 non-elite marathon runners pre- and post-marathon.

**Parameters**	**24 h**	**Immediately**	**48 h**	**F**	**SE**	**SE**	**SE**	**Fold-change**	**Fold-change**
	**Pre-race**	**Post-race**	**Post-race**	***p* value^a^**	***p* value**	***p* value**	***p* value**	**(2–1)**	**(3–1)**
	**(1)**	**(2)**	**(3)**		**(1–2)^b^**	**(1–3)^b^**	**(2–3)^b^**		
**PIINP (ng/ml)**									
Mean ± SD	9.05 ± 2.15	10.82 ± 3.44	11.00 ± 2.96	10.482	0.67	0.423	0.464	~1	~1
95% CI				0.001	0.053	0.001	1.000		
Lowerbound	7.94	9.05	9.48						
Upperbound	10.15	12.59	12.52						
**PIIANP (ng/ml)**									
Mean ± SD	2042.04 ± 567.25	1819.94 ± 548.34	1769.56 ± 419.67	3.151	101.824	129.449	132.059	~(–)1	~(–)1
95% CI				0.072	0.133	0.154	1.000		
Lowerbound	1750.38	1538.01	1553.79						
Upperbound	2333.69	2101.87	1985.33						
**C2C (ng/mL)**									
Mean ± SD	220.83 ± 39.50	188.67 ± 38.52	221.68 ± 45.64	8.989	7.778	6.713	8.604	~(–)1	~1
95% CI				0.003	0.002	1.000	0.004		
Lowerbound	200.52	168.87	198.21						
Upperbound	241.14	208.47	245.15						

a *Repeated measures ANOVA test*;

b *Bonferroni test; PIIANP, procollagen type II N terminal pro-peptide; C2C, cleavage type II collagen; PIINP, procollagen type II N terminal pro-peptide*.

### Correlation Between Biomarkers

Median sCK levels pre-race were positively correlated with sLDH pre-race (*r* = 0.758, *p* < 0.0001). Immediately post and 48-h post-race sCK median-circulating levels positively correlated with median sLDH immediately post (*r* = 0.623, *p* = 0.008) (*r* = 0.520, *p* = 0.033) and 48-h post-race (*r* = 0.730, *p* = 0.001) (*r* = 0.842, *p* < 0.0001).

Pre-race mean sPIINP-circulating levels positively correlated with median sCK 48-h post-race (*r* = 0.0494, *p* = 0.044) and with median sLDH immediately post-race (*r* = 0.616, *p* = 0.009). Mean sPIINP 48-h post-race levels positively correlated with median sLDH immediately post-race (*r* = 0.610, *p* = 0.009) and 48-h post-race (*r* = 0.488, *p* = 0.047).

**Figure 4:**
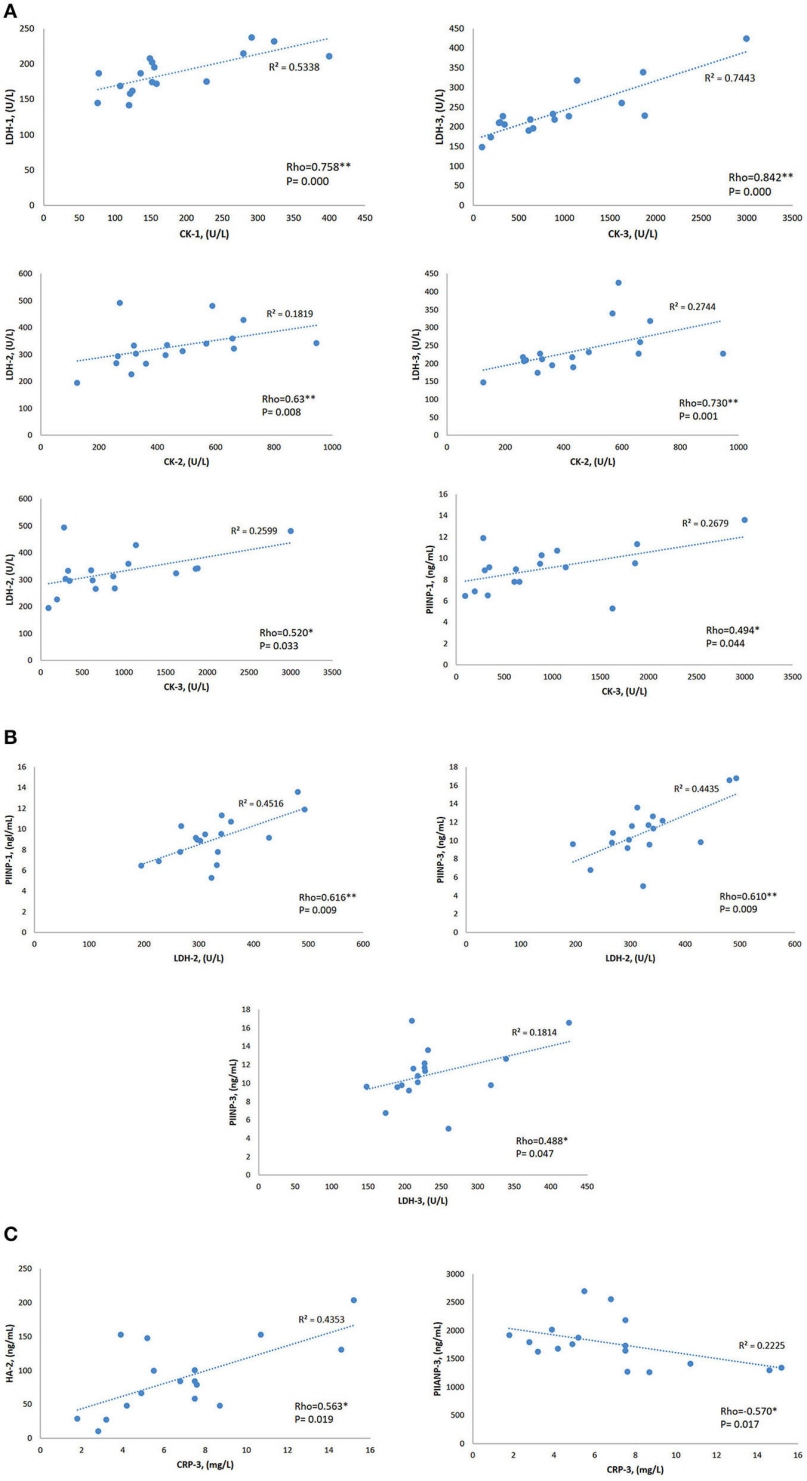
Rho Spearman's correlations and coefficient of linear regression *R*^2^ of the biomarkers that showed statistical significance variations. **(A)** CK Spearman's correlations. **(B)** LDH Spearman's correlations. **(C)** CRP Spearman's correlations. 1: pre-race; 2: post-race; 3: 48-h post-race. CK, creatine kinase; LDH, lactate dehydrogenase; CRP, C-reactive protein; PIINP, procollagen type II N terminal pro-peptide; PIIANP, procollagen type II N terminal pro-peptide. *p < 0.05, **p < 0.01.

Median sCRP 48-h post-race was positively correlated with mean sHA immediately post-race (*r* = 0.563, *p* = 0.019) and a negative correlation with mean sPIIANP 48-h post-race (*r* = −0.570, *p* = 0.017). Data is shown in Table 4 and Figure 4.

**Table 4:** Spearman's rho correlations [correlation coefficient and sig. (2-tailed)] between biomarkers.

**CK**			**LDH**		**CRP**	
**CK-1 vs. LDH-1**	**CK-2 vs. LDH-2**	**CK-3 vs. LDH-2**	**LDH-2 vs. PIINP-1**	**LDH-3 vs. PIINP-3**	**CRP-3 vs. HA-2**	**CRP-3 vs. PIIANP-3**
0.758^**^	0.623^**^	0.520^*^	0.616^**^	0.488^*^	0.563^*^	−0.570^*^
0.000	0.008	0.033	0.009	0.047	0.019	0.017
	**CK-2 vs. LDH-3**	**CK-3 vs. LDH-3**	**LDH-2 vs. PIINP-3**			
	0.730^**^	0.842^**^	0.610^**^			
	0.001	0.000	0.009			
		**CK-3 vs. PIINP-1**				
		0.494^*^				
		0.044				

^1^*pre-race; ^2^post-race; ^3^48-h post-race; CK, creatine kinase; LDH, lactate dehydrogenase; CRP, C-reactive protein; PIINP, procollagen type II N terminal pro-peptide; PIIANP, procollagen type II N terminal pro-peptide*.

* *p <0.05*;

** *p <0.01*.

## Discussion

Long-distance running has become very popular and as an exercise imposes high levels of stress on the musculoskeletal system (Neidhart et al., 2000). Nowadays, little is known on what is a physiological or expected acute inflammatory or cartilage metabolic response to an increase in physical activity (Cattano et al., 2017b; Driban et al., 2017).

This study broadens the set of serum cartilage biomarkers investigated (HA, CS846, YKL-40, PIINP, PIIANP, and C2C) for response to an intense exercise (Barcelona Marathon) in non-elite runners, with a special focus on the relation with inflammation and high-energy consumption. The major finding of this study is that marathon running modifies type II cartilage collagenous biomarkers serum levels with an increase in collagen synthesis (sPIINP increase) during 48 h and a transient decrease in collagen catabolism (sC2C decrease) immediately after race. Levels of sPIIANP and non-collagenous cartilage biomarkers (sCOMP, sCS846, and sYKL-40) remained relatively stable during the 48-h studied period. We found an increase in high-energy consumption (sCK and sLDH increase) and, in general, inflammatory reaction (sCRP increase) during 48 h correlated with collagen synthesis (sPIINP, sPIIANP) and an immediate post-marathon cartilage inflammatory and degradation protection mechanism (sHA increase).

A 3.4-fold increase in plasma hs-CRP has also been observed after day 1 in marathon runners (Kim et al., 2009); the increase returns to the pre-race level on day 4, with a dose-dependent behaviour reflected by a higher increase in ultra-marathon (200 km) runners. We observed even a higher median ten-fold increase of sCRP after day 2. CRP is a prototypic acute-phase protein produced in high quantities in the liver; however, the physiological function of CRP is still not completely understood (Peisajovich et al., 2008; Rhodes et al., 2011). Because CRP can function as an opsonin by coating particles and dying cells to facilitate their uptake by phagocytic cells (Gershov et al., 2000; Newling et al., 2019), we suggest that runners with higher-coating particles and cell damage produced during the intense exercise (Neidhart et al., 2000) will have higher levels of sCRP.

We agree with Kim et al. (2009) who described a temporary three-fold increase of CK in plasma during and after marathon running with further increase after day 1 that returns to pre-race levels after day 6. These authors demonstrated a dose-dependent behaviour of CK with a higher increase in ultra-marathon. We observed an even higher increase after day 2 (six-fold). These differences in behaviour may be due to distinct endurance training of runners (Wyss and Kaddurah-Daouk, 2000). Marathon running is an intense sport with high-energy demands, especially on musculoskeletal cells. Creatin kinase reaction is involved in the high-energy phosphate metabolism of cell in tissues with high-energy demands as muscle, brain, heart, and kidney. All CK isoenzymes catalyse the reversible transfer of the gamma-phosphate group of adenosine triphosphate (ATP) to the guanidino group of creatine (Cr) to yield adenosine diphosphate (ADP) and phosphoryl creatine (PCr) (Wyss and Kaddurah-Daouk, 2000).

Lactate dehydrogenase is a key glycolytic enzyme and is believed to be the major enzyme responsible for pyruvate to lactate conversion. It is released from many tissues into the circulation and indicates an increase in energy demands and in muscle cell membrane permeability, which occurs as the cell becomes energy depleted and by muscle cell damage, which occurs after prolonged exercise (Rumley et al., 1985). The increase in total serum LDH activity that occurs after marathon training can be modified by training (Rumley et al., 1985). We observed that this increase also occurs immediately after marathon running (two-fold increase) and stays elevated until 48-h post-race. LDH- and CK serum-level elevation correlated, especially at 48-h post-race; this suggests activated energy pathways or cell damage remains at least 48 h after intense physical activity.

HA is a biomarker specific to synovial joints and is the main component of articular cartilage. There is evidence that training (Cattano et al., 2017b) protocols may mitigate the HA biomarker response. HA binds to a cluster of different CD44 receptors, and this binding inhibits interleukin-1 (IL-1) expression and leads to a decline in matrix metalloproteinase (MMP) 1, 2, 3, 9, and 13 production (Karna et al., 2008). We observed a positive correlation with CRP that is consistent with a proposed protective effect on reduction of inflammation and on cartilage enhancement of proteoglycan synthesis (Moreland, 2003). Moreover, HA, lubricin, and phospholipid species endow synovial fluid with its viscoelastic properties and contribute either independently or together to the lubrication of the articular surfaces. Although a decrease in sHA concentration has been reported after 1-h walking (Pruksakorn et al., 2013), we observed a transient increase of sHA immediately after marathon running. Gordon et al. (2008) observed a transient increase after 1 h of monitored activity. These results suggest a different behaviour depending on intensity and duration of applied cyclic loads.

Cartilage oligomeric matrix protein is a component of the cartilage matrix synthesised by chondrocytes (Hedbom et al., 1992) and by fibroblast in tendon, meniscus, and synovial tissue (Hummel et al., 1998). It has been widely studied after exercise (Neidhart et al., 2000; Kersting et al., 2005; Mündermann et al., 2005; Kim et al., 2009; Niehoff et al., 2011; Celik et al., 2013), with various studies showing discrepancies in results, depending on varying study conditions. Serum COMP levels respond to mechanical stimulus, reportedly increase temporarily in a dose-dependent fashion in response to physical activity in highly trained marathon individuals and gradually return to baseline levels over 24 h (Neidhart et al., 2000; Kim et al., 2009); these increases are probably adaptive and mitigated by a running training protocol (Hoch et al., 2012; Celik et al., 2013). We find a trend for increased (n.s.) sCOMP levels immediately post-race, which was maintained 48 h after marathon running in well-trained non-elite runners. It has been suggested that serum COMP levels after physical activity that reflects enhanced diffusion of COMP fragments form cartilage to blood due to joint loading (Mündermann et al., 2005) or increase in lymphatic drainage (Neidhart et al., 2000; Kersting et al., 2005). The higher baseline serum levels observed in marathon runners, in osteoarthritis, and rheumatoid arthritis than in healthy controls (Neidhart et al., 2000; Garnero et al., 2001; Sharif et al., 2007) and the larger lag time to return to basal levels after a marathon (Neidhart et al., 2000; Kim et al., 2009) than after a moderate physical activity (Mündermann et al., 2005) suggest a maintained increase in synthesis or diffusion due to the rapid elimination of COMP once it has reached the circulation (Andersson et al., 2006).

Kersting et al. (2005) found no COMP increase 25 min or 2.5 h after a 1-h run at a self-selected pace in physically trained individuals. The different behaviours of serum COMP levels may be explained by their sensitivity to the loading mode, for instance, slow and deep knee bends do not affect COMP levels (Niehoff et al., 2010) and by the adaptations that may be experiment within the time frame of load application (Liphardt et al., 2018) and with training (Hoch et al., 2012; Celik et al., 2013).

Serum YKL-40 is a glycoprotein secreted by chondrocyte (Einarsson et al., 2013) and also expressed in osteoblast and osteocytes present in osteophytes (Živanović et al., 2009). It has been hypothesised that YKL-40 production is a part of the inflammatory response in chondrocytes, acting to limit connective tissue degradation (Johansen et al., 2001) and linked with tissue remodelling, joint injury, and “*in situ*” inflammatory macrophages (Lorenz et al., 2005). Our findings also suggest that sYKL-40 is not associated with sCRP (Karalilova et al., 2018). Serum CS846, an aggrecan synthesis marker, was not significantly changed measures of 0.5 and 5.5 h after 30 min walk, whereas an increase in the synthesis correlates with MRI cartilage thickening in patients with medial knee OA and correlates with an increase in collagen degradation biomarker C1, C2 (Chu et al., 2018). We observed a similar behaviour of sCOMP, sCS846, andYKL-40 with no significant changes in serum levels after marathon running, but with a tendency of sCOMP to increase and of CS846 to decrease. The increase of sYKL-40 and sCS846, reportedly, protects against cartilage degradation (Johansen et al., 2001; Chu et al., 2018). The absence of changes in sYKL-40 levels and the tendency to decrease of CS846 are consistent with the absence of an increase of collagen degradation (sC2C decrease) that we observed after marathon running and may indicate no increase in diffusion, or cartilage matrix turnover or damage (Otterness et al., 2000).

Collagen is produced and secreted as a precursor molecule with C- and N-terminal extensions termed procollagen peptides (PIICP and PIINP). Since procollagen peptides are released from the parent molecule in exact proportions, the concentration of these peptides assesses the biosynthetic activity (Munk et al., 2014). In the extracellular space, the procollagen terminal extensions are cleaved off by specific C- and N-pro-peptidases; after which, the type II collagen through a collagen-proteoglycan interaction can participate in fibril formation (Graham et al., 2000). N-terminal pro-peptide of type II collagen (PIINP) is a biomarker-reflecting cartilage formation. PIINP exists in two main splice variants termed as type IIA and type IIB collagen NH2-propeptide (PIIANP, PIIBNP) (Nah and Upholt, 1991). PIIANP has been recognised as a cartilage formation biomarker, probably restricted to embryogenesis and fracture healing but may be reexpressed in osteoarthritis (Aigner et al., 1999). On the other hand, PIIBNP is mainly expressed during type II collagen formation in healthy adult cartilage (Sandell et al., 1991). There is, reportedly, no correlation between PIIBNP and PIIANP levels in knee osteoarthritis, rheumatoid arthritis, and paediatric serum samples (Luo et al., 2018). Serum-PIIANP is, reportedly, decreased in patients with knee osteoarthritis (Sharif et al., 2007) and decreased in rheumatoid arthritis of long duration (Rousseau et al., 2004).

The increase of sPIINP we observed after marathon running was maintained over 48 h. This may reflect type II collagen formation in healthy cartilage (PIIBNP) because the splice variant PIIANP showed a trend to decrease after marathon running. Healthy cartilage is characterised by a low turnover of collagens (Tchetina, 2011), but upregulation of col II and col I synthesis in chondrocytes has been reported in response to cyclic compression *in vitro* with a time constant of 2.9 h (Bonassar et al., 2001), a finding consistent with our *in vivo* observations in marathon running.

The cleavage of type II collagen by collagenases yields fragments, such as the C2C epitope, and serum levels of C2C correlate with cartilage degeneration in patients with symptomatic knee OA (King et al., 2004). We observed a transient decrease in sC2C levels immediately after marathon running that returns to normal 48 h later, possibly indicating cyclic loads transiently reduce collagen catabolism by collagenases. Our results on collagen cartilage biomarkers metabolism (sPIINP increase and sC2C decrease) agree with those of Azukizawa (Azukizawa et al., 2019) who observed well-rounded exercise may have beneficial effects on type II collagen metabolism, especially in people without OA increasing sPIICP, secreted in exact proportions than PIINP, and decreasing urine-C2C and uCTX-II (urine carboxy-terminal crosslinked telopeptide of type II collagen).

The positive correlation between sPIINP and sCK and sLDH levels and the negative correlation between sCRP and sPIIANP may indicate an interaction of type II collagen synthesis in adult cartilage with the high-energy demands required and the inflammatory reaction produced after marathon running. The observed changes in the studied biomarkers appear to be caused by altered metabolic activity rather than diffusion due to marathon because of the large duration (3–4 h) of marathon stimulus enough for cell metabolism activation (Bonassar et al., 2001) and the time lag (48 h) that biomarkers changes persist after the stimulus (Erhart-Hledik et al., 2012).

This study has the following limitations. First, we had a limited sample size with no control group, but, even with a small sample size, we found statistically significant differences. The aim of the study was to know whether there are changes in the biomarker profile in healthy subjects after undergoing high-intensity exercise, a marathon. We considered that the control group would be made of a sample of healthy individuals who do not run the marathon, which, in this case, will be the baseline or pre-race point. Second, we did document the diet and exercise constraints by the participants in the 48-h period pre and post-marathon, but we did not have the runners controlled in a sport centre under strict supervision 48 h before and after the marathon, and that can be a limitation for the strict follow-up of diet and exercise constraints by runners. Third, imaging exams on joint status (knee, hips, and ankles) were not evaluated before or after the study; presence of osteoarthritis can alter biomarkers levels and behaviour, although no participants had symptoms or previous surgery on lower limb joints. Fourth, we selected specific inflammatory, energy enzymes, non-collagenous, and type II collagenous cartilage biomarkers; other biomarkers may show different patterns, but we believe studied biomarkers well represent cartilage metabolism. Fifth, a singlet data analysis of the biomarkers may be considered a limitation as duplicate or triplicate analysis has been the standard practise in research but not in clinical diagnostic labs. Nowadays, it is questionable if duplicate analysis improves variability, and individual analysis could be adopted as standard practise in modern ligand-binding assays (Ye et al., 2018). Sixth, corrections for plasma volume change that can occur following an acute bout of exercise has been recommended when studying serum biomarkers (Kargotich et al., 1997); however, this does not apply to all published studies (Neidhart et al., 2000; Kersting et al., 2005; Mündermann et al., 2005; Niehoff et al., 2011). Immediate and 48-h post-marathon plasma volume change determined with haemoglobin and haematocrit data of a previous study of our group (Roca et al., 2019) in 41 runners of 2016 Barcelona Marathon was −1.2 and −1.7%, respectively. The percentage of biomarker change when correcting for plasma volume is small, and we believe it would apply to our study.

In conclusion, we confirmed previous studies suggesting marathon running is an exercise with high-energy demands (sCK and sLDH levels increase) that provokes a high and durable general inflammatory reaction (sCRP levels increase). We observed a transient increase in sHA levels immediately post-marathon that may be a mechanism to protect inflammation and cartilage, accompanied by an upregulation of type II collagen cartilage metabolism increasing fibrils synthesis (sPIINP levels increase) and decreasing its catabolism (sC2C levels decrease), without changes in non-collagenous cartilage metabolism (sCOMP, sCS846, and sYKL-40). The duration of marathon stimulus and the lag time that serum levels changes persist suggest that changes in the studied biomarkers are caused by metabolic activity rather than diffusion. Metabolic changes on sHA and sPIINP synthesis may be related to energy consumption (sCK, sLDH) and the inflammatory reaction (sCRP) produced.

## Data Availability Statement

The raw data supporting the conclusions of this article will be made available by the authors, without undue reservation.

## Ethics Statement

The studies involving human participants were reviewed and approved by Germans Trias i Pujol University Hospital Ethics Committee. The patients/participants provided their written informed consent to participate in this study.

## Author Contributions

JH-H participated in the conception and design of the study, analysis, interpretation of data, and drafting the article. LN participated in acquisition of data, statistical analysis, and interpretation of data. ER participated in the provision of study patients, in technical and logistic support, in acquisition of data, and in collection and assembly of data. ER-L participated in technical and logistic support, in acquisition of data, and in collection and assembly of data. JA and AB-G participated in technical and logistic support and critical revision of the article. All authors read and approved the final version to be submitted.

## Acknowledgements

We thank A. Cserkóová for the technical processing in the laboratory.

## Conflict of Interest

The authors declare that the research was conducted in the absence of any commercial or financial relationships that could be construed as a potential conflict of interest.

## Publisher's Note

All claims expressed in this article are solely those of the authors and do not necessarily represent those of their affiliated organizations, or those of the publisher, the editors and the reviewers. Any product that may be evaluated in this article, or claim that may be made by its manufacturer, is not guaranteed or endorsed by the publisher.

## References

[B1] Aigner T. Zhu Y. Chansky H. H. Matsen F. A. Maloney W. J. Sandell L. J. (1999). Reexpression of type IIA procollagen by adult articular chondrocytes in osteoarthritic cartilage. Arthritis Rheum. 42, 1443–1450. 10.1002/1529-0131(199907)42:7 <1443::AID-ANR18>3.0.CO;2-A 10403272

[B2] Andersson M. L. E. Petersson I. F. Karlsson K. E. Jonsson E. N. Månsson B. Heinegård D.. (2006). Diurnal variation in serum levels of cartilage oligomeric matrix protein in patients with knee osteoarthritis or rheumatoid arthritis. Ann. Rheum. Dis. 65, 1490–1494. 10.1136/ard.2005.051292 16707535 PMC1798358

[B3] Azukizawa M. Ito H. Hamamoto Y. Fujii T. Morita Y. Okahata A.. (2019). The effects of well-rounded exercise program on systemic biomarkers related to cartilage metabolism. Cartilage 10, 451–458. 10.1177/1947603518767998 29644876 PMC6755879

[B4] Bonassar L. J. Grodzinsky A. J. Frank E. H. Davila S. G. Bhaktav N. R. Trippel S. B. (2001). The effect of dynamic compression on the response of articular cartilage to insulin-like growth factor-I. J. Orthop. Res. 19, 11–17. 10.1016/S0736-0266(00)00004-8 11332605

[B5] Boocock M. McNair P. Cicuttini F. Stuart A. Sinclair T. (2009). The short-term effects of running on the deformation of knee articular cartilage and its relationship to biomechanical loads at the knee. Osteoarthr. Cartil. 17, 883–890. 10.1016/j.joca.2008.12.010 19246217

[B6] Cattano N. M. Driban J. B. Barbe M. F. Tierney R. Amin M. Sitler M. R. (2017a). Physical activity levels and quality of life relate to collagen turnover and inflammation changes after running. J. Orthop. Res. 35, 612–617. 10.1002/jor.23250 27035929

[B7] Cattano N. M. Driban J. B. Cameron K. L. Sitler M. R. (2017b). Impact of physical activity and mechanical loading on biomarkers typically used in osteoarthritis assessment: current concepts and knowledge gaps. Ther. Adv. Musculoskelet. Dis. 9, 11–21. 10.1177/1759720X16670613 28101145 PMC5228638

[B8] Celik O. Salci Y. Ak E. Kalaci A. Korkusuz F. (2013). Serum cartilage oligomeric matrix protein accumulation decreases significantly after 12weeks of running but not swimming and cycling training—a randomised controlled trial. Knee 20, 19–25. 10.1016/j.knee.2012.06.001 22770506

[B9] Chu C. R. Sheth S. Erhart-Hledik J. C. Do B. Titchenal M. R. Andriacchi T. P. (2018). Mechanically stimulated biomarkers signal cartilage changes over 5 years consistent with disease progression in medial knee osteoarthritis patients. J. Orthop. Res. 36, 891–897. 10.1002/jor.23720 28862360 PMC6548432

[B10] Di Rosa M. Malaguarnera G. De Gregorio C. Drago F. Malaguarnera L. (2013). Evaluation of CHI3L-1 and CHIT-1 expression in differentiated and polarized macrophages. Inflammation 36, 482–492. 10.1007/s10753-012-9569-8 23149946

[B11] Doré D. A. Winzenberg T. M. Ding C. Otahal P. Pelletier J. P. Martel-Pelletier J.. (2013). The association between objectively measured physical activity and knee structural change using MRI. Ann. Rheum. Dis. 72, 1170–1175. 10.1136/annrheumdis-2012-201691 22896739

[B12] Driban J. B. Hootman J. M. Sitler M. R. Harris K. P. Cattano N. M. (2017). Is participation in certain sports associated with knee osteoarthritis? A systematic review. J. Athl. Train. 52, 497–506. 10.4085/1062-6050-50.2.08 25574790 PMC5488840

[B13] Einarsson J. M. Bahrke S. Sigurdsson B. T. Ng C. H. Petersen P. H. Sigurjonsson O. E.. (2013). Partially acetylated chitooligosaccharides bind to YKL-40 and stimulate growth of human osteoarthritic chondrocytes. Biochem. Biophys. Res. Commun. 434, 298–304. 10.1016/j.bbrc.2013.02.122 23541584

[B14] Erhart-Hledik J. C. Favre J. Asay J. L. Smith R. L. Giori N. J. Mündermann A.. (2012). A relationship between mechanically-induced changes in serum cartilage oligomeric matrix protein (COMP) and changes in cartilage thickness after 5 years. Osteoarthr. Cartil. 20, 1309–1315. 10.1016/j.joca.2012.07.018 22868052

[B15] Garnero P. Piperno M. Gineyts E. Christgau S. Delmas P. D. Vignon E. (2001). Cross sectional evaluation of biochemical markers of bone, cartilage, and synovial tissue metabolism in patients with knee osteoarthritis: relations with disease activity and joint damage. Ann. Rheum. Dis. 60, 619–626. 10.1136/ard.60.6.619 11350852 PMC1753666

[B16] Gershov D. Kim S. J. Brot N. Elkon K. B. (2000). C-reactive protein binds to apoptotic cells, protects the cells from assembly of the terminal complement components, and sustains an antiinflammatory innate immune response: Implications for systemic autoimmunity. J. Exp. Med. 192, 1353–1363. 10.1084/jem.192.9.1353 11067883 PMC2193350

[B17] Gordon C. D. Stabler S. T. Kraus V. B. (2008). Variation in osteoarthritis biomarkers from activity not food consumption. Clin. Chim. Acta 398, 21–26. 10.1016/j.cca.2008.07.031.Variation 18727924 PMC2586038

[B18] Graham H. K. Holmes D. F. Watson R. B. Kadler K. E. (2000). Identification of collagen fibril fusion during vertebrate tendon morphogenesis. The process relies on unipolar fibrils and is regulated by collagen-proteoglycan interaction. J. Mol. Biol. 295, 891–902. 10.1006/jmbi.1999.3384 10656798

[B19] Hedbom E. Antonsson P. Hjerpe A. Aeschlimann D. Paulsson M. Rosa-Pimentel E.. (1992). Cartilage matrix proteins. An acidic oligomeric protein (COMP) detected only in cartilage. J. Biol. Chem. 267, 6132–6136. 10.1016/s0021-9258(18)42671-3 1556121

[B20] Hoch J. M. Mattacola C. G. Bush H. M. Medina McKeon J. M. Hewett T. E. Lattermann C. (2012). Longitudinal documentation of serum cartilage oligomeric matrix protein and patient-reported outcomes in collegiate soccer athletes over the course of an athletic season. Am. J. Sports Med. 40, 2583–2589. 10.1177/0363546512458260 22967826 PMC3615716

[B21] Hummel K. M. Neidhart M. Vilim V. Hauser N. Aicher W. K. Gay R. E.. (1998). Analysis of cartilage oligomeric matrix protein (COMP) in synovial fibroblasts and synovial fluids. Br. J. Rheumatol. 37, 721–728. 10.1093/rheumatology/37.7.721 9714346

[B22] Johansen J. S. Olee T. Price P. A. Hashimoto S. Ochs R. L. Lotz M. (2001). Regulation of YKL-40 production by human articular chondrocytes. Arthritis Rheum. 44, 826–837. 10.1002/1529-0131(200104)44:4 <826::AID-ANR139>3.0.CO;2-U 11315922

[B23] Jordan J. M. Luta G. Stabler T. Renner J. B. Dragomir A. D. Vilim V.. (2003). Ethnic and sex differences in serum levels of cartilage oligomeric matrix protein: The johnston county osteoarthritis project. Arthritis Rheum. 48, 675–681. 10.1002/art.10822 12632420

[B24] Karalilova R. Kazakova M. Batalov A. Sarafian V. (2018). Correlation between protein YKL-40 and ultrasonographic findings in active knee osteoarthritis. Med. Ultrason. 20, 57–63. 10.11152/mu-1247 29400369

[B25] Kargotich S. Goodman C. Keast D. Fry R. W. Garcia-Webb P. Crawford P. M.. (1997). Influence of exercise-induced plasma volume changes on the interpretation of biochemical data following high-intensity exercise. Clin. J. Sport Med. 7, 185–191. 9262885 10.1097/00042752-199707000-00006

[B26] Karna E. Miltyk W. Surazyński A. Pałka J. A. (2008). Protective effect of hyaluronic acid on interleukin-1-induced deregulation of βeta 1 -integrin and insulin-like growth factor-I receptor signaling and collagen biosynthesis in cultured human chondrocytes. Mol. Cell. Biochem. 308, 57–64. 10.1007/s11010-007-9612-5 17899316

[B27] Kersting U. G. Stubendorff J. J. Schmidt M. C. Brüggemann G. P. (2005). Changes in knee cartilage volume and serum COMP concentration after running exercise. Osteoarthr. Cartil. 13, 925–934. 10.1016/j.joca.2005.06.005 16154364

[B28] Kim H. J. Lee Y. H. Kim C. K. (2009). Changes in serum cartilage oligomeric matrix protein (COMP), plasma CPK and plasma hs-CRP in relation to running distance in a marathon (42.195 km) and an ultra-marathon (200 km) race. Eur. J. Appl. Physiol. 105, 765–770. 10.1007/s00421-008-0961-x 19125286

[B29] King K. B. Lindsey C. T. Dunn T. C. Ries M. D. Steinbach L. S. Majumdar S. (2004). A study of the relationship between molecular biomarkers of joint degeneration and the magnetic resonance-measured characteristics of cartilage in 16 symptomatic knees. Magn. Reson. Imaging 22, 1117–1123. 10.1016/j.mri.2004.08.001 15527998

[B30] Liphardt A. M. Mündermann A. Andriacchi T. P. Achtzehn S. Heer M. Mester J. (2018). Sensitivity of serum concentration of cartilage biomarkers to 21-days of bed rest. J. Orthop. Res. 36, 1465–1471. 10.1002/jor.23786 29077223

[B31] Liphardt A. M. Mündermann A. Koo S. Bäcker N. Andriacchi T. P. Zange J.. (2009). Vibration training intervention to maintain cartilage thickness and serum concentrations of cartilage oligometric matrix protein (COMP) during immobilization. Osteoarthr. Cartil. 17, 1598–1603. 10.1016/j.joca.2009.07.007 19747585

[B32] Lorenz H. Wenz W. Ivancic M. Steck E. Richter W. (2005). Early and stable upregulation of collagen type II, collagen type I and YKL40 expression levels in cartilage during early experimental osteoarthritis occurs independent of joint location and histological grading. Arthritis Res. Ther. 7, 1–10. 10.1186/ar1471 15642136 PMC1064896

[B33] Luo Y. He Y. Reker D. Gudmann N. S. Henriksen K. Simonsen O.. (2018). A novel high sensitivity type II collagen blood-based biomarker, PRO-C2, for assessment of cartilage formation. Int. J. Mol. Sci. 19, 1–15. 10.3390/ijms19113485 30404167 PMC6275061

[B34] Maqueda M. Roca E. Brotons D. Soria J. M. Perera A. (2017). Affected pathways and transcriptional regulators in gene expression response to an ultra-marathon trail: Global and independent activity approaches. PLoS ONE 12, 1–26. 10.1371/journal.pone.0180322 29028836 PMC5640184

[B35] Matt Denning W. Winward J. G. Pardo M. B. Hopkins J. T. Seeley M. K. (2015). Body weight independently affects articular cartilage catabolism. J. Sport. Sci. Med. 14, 290–296. 25983577 PMC4424457

[B36] Moreland L. W. (2003). Intra-articular hyaluronan (hyaluronic acid) and hylans for the treatment of osteoarthritis: mechanisms of action. Arthritis Res. Ther. 5, 54–67. 10.1186/ar623 12718745 PMC165033

[B37] Mündermann A. Dyrby C. O. Andriacchi T. P. King K. B. (2005). Serum concentration of cartilage oligomeric matrix protein (COMP) is sensitive to physiological cyclic loading in healthy adults. Osteoarthr. Cartil. 13, 34–38. 10.1016/j.joca.2004.09.007 15639635

[B38] Munk H. L. Svendsen A. J. Hjelmborg J. v B Sorensen G. L. Kyvik K. O.. (2014). Heritability assessment of cartilage metabolism. A twin study on circulating procollagen IIA N-terminal propeptide (PIIANP). Osteoarthr. Cartil. 22, 1142–1147. 10.1016/j.joca.2014.06.027 25008205

[B39] Nah H. D. Upholt W. B. (1991). Type II collagen mRNA containing an alternatively spliced exon predominates in the chick limb prior to chondrogenesis. J. Biol. Chem. 266, 23446–23452. 1744138

[B40] Neidhart M. Müller-Ladner U. Frey W. Bosserhoff A. K. Colombani P. C. Frey-Rindova P.. (2000). Increased serum levels of non-collagenous matrix proteins (cartilage oligomeric matrix protein and melanoma inhibitory activity) in marathon runners. Osteoarthr. Cartil. 8, 222–229. 10.1053/joca.1999.0293 10806050

[B41] Nescolarde L. Roca E. Bogónez-Franco P. Hernández-Hermoso J. Bayes-Genis A. Ara J. (2020). Relationship between bioimpedance vector displacement and renal function after a marathon in non-elite runners. Front. Physiol. 11:352. 10.3389/fphys.2020.00352 32435201 PMC7218173

[B42] Newling M. Sritharan L. van der Ham A. J. Hoepel W. Fiechter R. H. de Boer L.. (2019). C-reactive protein promotes inflammation through FcγR-induced glycolytic reprogramming of human macrophages. J. Immunol. 203, 225–235. 10.4049/jimmunol.1900172 31118224

[B43] Niehoff A. Kersting U. G. Helling S. Dargel J. Maurer J. Thevis M.. (2010). Different mechanical loading protocols influence serum cartilage oligomeric matrix protein levels in young healthy humans. Eur. J. Appl. Physiol. 110, 651–657. 10.1007/s00421-010-1529-0 20544356

[B44] Niehoff A. Müller M. Brüggemann L. Savage T. Zaucke F. Eckstein F.. (2011). Deformational behaviour of knee cartilage and changes in serum cartilage oligomeric matrix protein (COMP) after running and drop landing. Osteoarthr. Cartil. 19, 1003–1010. 10.1016/j.joca.2011.04.012 21616158

[B45] Otterness I. G. Swindell A. C. Zimmerer R. O. Poole A. R. Ionescu M. Weiner E. (2000). An analysis of 14 molecular markers for monitoring osteoarthritis: Segregation of the markers into clusters and distinguishing osteoarthritis at baseline. Osteoarthr. Cartil. 8, 180–185. 10.1053/joca.1999.0288 10806045

[B46] Peisajovich A. Marnell L. Mold C. Du Clos T. W. (2008). C-reactive protein at the interface between innate immunity and inflammation. Expert Rev. Clin. Immunol. 4, 379–390. 10.1586/1744666X.4.3.379 20476927

[B47] Pruksakorn D. Tirankgura P. Luevitoonvechkij S. Chamnongkich S. Sugandhavesa N. Leerapun T.. (2013). Changes in the serum cartilage biomarker levels of healthy adults in response to an uphill walk. Singapore Med. J. 54, 702–708. 10.11622/smedj.2013245 24356757

[B48] Radzimiński Ł. Jastrzebski Z. López-Sánchez G. F. Szwarc A. Duda H. Stuła A.. (2020). Relationships between training loads and selected blood parameters in professional soccer players during a 12-day sports camp. Int. J. Environ. Res. Public Health 17, 1–10. 10.3390/ijerph17228580 33227932 PMC7699258

[B49] Rhodes B. Fürnrohr B. G. Vyse T. J. (2011). C-reactive protein in rheumatology: Biology and genetics. Nat. Rev. Rheumatol. 7, 282–289. 10.1038/nrrheum.2011.37 21468143

[B50] Roca E. Cantó E. Nescolarde L. Perea L. Bayes-Genis A. Sibila O.. (2019). Effects of a polysaccharide-based multi-ingredient supplement on salivary immunity in non-elite marathon runners. J. Int. Soc. Sports Nutr. 16, 1–3. 10.1186/s12970-019-0281-z 30909945 PMC6434855

[B51] Roca E. Nescolarde L. Lupón J. Barallat J. Januzzi J. L. Liu P.. (2017). The dynamics of cardiovascular biomarkers in non-elite marathon runners. J. Cardiovasc. Transl. Res. 10, 206–208. 10.1007/s12265-017-9744-2 28382580

[B52] Rousseau J. C. Zhu Y. Miossec P. Vignon E. Sandell L. J. Garnero P.. (2004). Serum levels of type IIA procollagen amino terminal propeptide (PIIANP) are decreased in patients with knees osteoarthritis and rheumatoid arthritis. Osteoarthr. Cartil. 12, 440–447. 10.1016/j.joca.2004.02.004 15135140

[B53] Rumley A. G. Pettigrew A. R. Colgan M. E. Taylor R. Grant S. Manzie A.. (1985). Serum lactate dehydrogenase and creatine kinase during marathon training. Br. J. Sports Med. 19, 152–155. 10.1136/bjsm.19.3.152 4075065 PMC1478243

[B54] Sandell L. J. Morris N. Robbins J. R. Goldring M. B. (1991). Alternatively spliced type II procollagen mRNAs define distinct populations of cells during vertebral development: Differential expression of the amino-propeptide. J. Cell Biol. 114, 1307–1319. 10.1083/jcb.114.6.1307 1894696 PMC2289128

[B55] Sharif M. Kirwan J. Charni N. Sandell L. J. Whittles C. Garnero P. (2007). A 5-yr longitudinal study of type IIA collagen synthesis and total type II collagen degradation in patients with knee osteoarthritis—association with disease progression. Rheumatology 46, 938–943. 10.1093/rheumatology/kel409 17387119

[B56] Tchetina E. V. (2011). Developmental mechanisms in articular cartilage degradation in osteoarthritis. Arthritis 2011, 1–16. 10.1155/2011/683970 22046522 PMC3199933

[B57] Wyss M. Kaddurah-Daouk R. (2000). Creatine and creatinine metabolism. Physiol. Rev. 80, 1107–1213. 10.1152/physrev.2000.80.3.1107 10893433

[B58] Ye Z. Tu J. Midde K. Edwards M. Bennett P. (2018). Singlicate analysis: Should this be the default for biomarker measurements using ligand-binding assays? Bioanalysis 10, 909–912. 10.4155/bio-2018-0067 29923752

[B59] Živanović S. Rackov L. P. Vojvodić D. Vučetić D. (2009). Human cartilage glycoprotein 39-biomarker of joint damage in knee osteoarthritis. Int. Orthop. 33, 1165–1170. 10.1007/s00264-009-0747-8 19308408 PMC2898969

