# Supplementary material for: Marathon Running Increases Synthesis and Decreases Catabolism of Joint Cartilage Type II Collagen Accompanied by High-Energy Demands and an Inflamatory Reaction
Source: Front Physiol. 2021 Oct 11;12:722718. doi: 10.3389/fphys.2021.722718 (PMC8542987; doi:10.3389/fphys.2021.722718)
Supplement: Supplementary file 1 [file Data_Sheet_1.PDF]

ID	Race-time (hh:mm:ss)	Age	Hight	W_1	CK_1	CK_2	CK_3
1	3:13:53	49	1,80	78,5	323	568	1862
2	3:11:51	35	1,76	65,3	77	271	282
3	3:28:20	41	1,70	75,5	152	260	890
4	3:54:17	42	1,79	79,9	121	662	1628
5	3:43:40	41	1,86	81,6	400	434	606
6	3:29:51	43	1,82	77,7	280	487	871
7	2:58:24	43	1,87	83,2	152	320	327
8	3:40:40	43	1,78	80,6	120	946	1882
9	3:29:56	42	1,77	68,2	155	311	194
10	3:14:32	36	1,78	67,3	124	429	623
11	3:38:08	40	1,76	81,8	107	327	296
12	3:19:07	41	1,84	87,0	149	589	2999
13	3:45:40	44	1,83	86,7	76	361	658
14	3:37:46	46	1,92	90,1	291	696	1140
15	3:05:34	38	1,83	76,8	228	657	1051
16	3:59:30	41	1,71	80,3	136	265	344
17	3:32:05	37	1,71	74,7	158	125	95

PerChCK2_1	PerChCK3_1	PerChCK3_2	LDH_1	LDH_2	LDH_3	PerChLDH2_1
75,9	476,5	227,8	232	341	339	47,0
251,9	266,2	4,1	187	493	210	163,6
71,1	485,5	242,3	174	268	218	54,0
447,1	1245,5	145,9	158	323	260	104,4
8,5	51,5	39,6	211	335	190	58,8
73,9	211,1	78,9	215	312	232	45,1
110,5	115,1	2,2	202	333	227	64,9
688,3	1468,3	98,9	142	342	228	140,8
100,6	25,2	-37,6	195	227	174	16,4
246,0	402,4	45,2	162	297	218	83,3
205,6	176,6	-9,5	169	303	212	79,3
295,3	1912,8	409,2	208	481	425	131,3
375,0	765,8	82,3	145	266	196	83,4
139,2	291,8	63,8	237	428	318	80,6
188,2	361,0	60,0	175	359	227	105,1
94,9	152,9	29,8	187	295	206	57,8
-20,9	-39,9	-24,0	172	195	148	13,4

**PerChLDH3_1**	**PerChLDH3_2**	CRP_1	CRP_2	CRP_3	**PerChCRP_1**	**PerChCRP3_1**
46,1	-0,6	1,0	0,7	3,9	-30,0	290,0
12,3	-57,4	0,9	0,3	7,5	-66,7	733,3
25,3	-18,7	0,4	0,5	4,2	25,0	950,0
64,6	-19,5	0,2	0,4	14,6	100,0	7200,0
-10,0	-43,3	1,2	0,4	4,9	-66,7	308,3
7,9	-25,6	0,6	0,3	5,5	-50,0	816,7
12,4	-31,8	1,3	1,7	7,5	30,8	476,9
60,6	-33,3	0,7	0,6	7,5	-14,3	971,4
-10,8	-23,3	0,6	0,7	3,2	16,7	433,3
34,6	-26,6	0,3	0,3	5,2	0,0	1633,3
25,4	-30,0	1,0	1,0	7,6	0,0	660,0
104,3	-11,6	0,6	0,4	2,8	-33,3	366,7
35,2	-26,3	1,2	1,5	15,2	25,0	1166,7
34,2	-25,7	0,7	0,3	8,7	-57,1	1142,9
29,7	-36,8	1,9	1,2	10,7	-36,8	463,2
10,2	-30,2	0,7	0,5	6,8	-28,6	871,4
-14,0	-24,1	3,4	0,8	1,8	-76,5	-47,1

PerChCRP3_2	HA_1	HA_2	HA_3	PerChHA2_1	PerChHA3_1	PerChHA3_2	COMP_1
457,1	29,07	152,99	16,28	426,3	-44,0	-89,4	223,97
2400,0	14,16	83,98	18,85	493,1	33,1	-77,6	297,38
740,0	11,79	47,82	12,05	305,6	2,2	-74,8	215,20
3550,0	17,91	130,40	35,30	628,1	97,1	-72,9	289,20
1125,0	12,50	66,45	23,77	431,6	90,2	-64,2	238,58
1733,3	12,36	99,37	23,68	704,0	91,6	-76,2	276,13
341,2	15,75	100,25	11,11	536,5	-29,5	-88,9	262,68
1150,0	29,29	58,36	13,82	99,2	-52,8	-76,3	151,38
357,1	13,64	27,78	15,16	103,7	11,1	-45,4	106,82
1633,3	36,61	147,89	19,30	304,0	-47,3	-86,9	305,30
660,0	21,18	79,24	19,14	274,1	-9,6	-75,8	241,75
600,0	12,69	10,52	13,79	-17,1	8,7	31,1	226,17
913,3	25,15	203,63	20,63	709,7	-18,0	-89,9	134,00
2800,0	19,74	48,16	25,11	144,0	27,2	-47,9	234,86
791,7	16,84	152,47	26,59	805,4	57,9	-82,6	190,76
1260,0	32,73	84,19	41,44	157,2	26,6	-50,8	182,67
125,0	24,43	28,65	20,89	17,3	-14,5	-27,1	197,80

COMP_2	COMP3	**PerChCOMP2_1**	**PerChCOMP3_1**	**PerChCOMP3_2**	CS846_1	CS846_2
233,43	251,67	4,2	12,4	7,8	106,10	99,71
278,65	263,86	-6,3	-11,3	-5,3	78,93	86,92
280,30	187,99	30,3	-12,6	-32,9	93,84	96,79
300,65	277,35	4,0	-4,1	-7,7	49,12	71,45
212,26	239,94	-11,0	0,6	13,0	96,87	125,92
246,51	205,99	-10,7	-25,4	-16,4	93,19	79,90
231,66	191,46	-11,8	-27,1	-17,4	92,22	99,81
178,04	259,39	17,6	71,4	45,7	148,15	156,56
124,96	147,49	17,0	38,1	18,0	65,85	67,73
277,19	264,80	-9,2	-13,3	-4,5	165,34	90,54
285,56	311,45	18,1	28,8	9,1	38,93	31,73
249,68	246,99	10,4	9,2	-1,1	34,31	44,95
140,73	128,97	5,0	-3,8	-8,4	69,99	92,81
276,46	246,67	17,7	5,0	-10,8	63,50	49,78
158,91	297,57	-16,7	56,0	87,3	32,49	69,61
208,82	198,62	14,3	8,7	-4,9	45,60	67,36
284,46	217,91	43,8	10,2	-23,4	89,83	7,27

CS846_3	**PerChCS846_2_1**	**PerChCS846_3_1**	**PerChCS846_3_2**	YKL40_1	YKL40_2	YKL40_3
91,35	-6,0	-13,9	-8,4	3,92	4,22	3,93
88,34	10,1	11,9	1,6	4,28	4,01	3,99
84,22	3,1	-10,3	-13,0	5,89	5,50	5,24
104,47	45,5	112,7	46,2	3,89	5,16	5,16
127,59	30,0	31,7	1,3	12,38	11,12	20,59
98,52	-14,3	5,7	23,3	3,54	3,75	3,49
123,39	8,2	33,8	23,6	4,11	4,01	3,74
89,81	5,7	-39,4	-42,6	3,13	2,83	2,41
77,11	2,9	17,1	13,8	4,22	4,68	4,79
69,63	-45,2	-57,9	-23,1	2,37	2,43	2,46
20,42	-18,5	-47,5	-35,6	2,71	2,69	2,39
36,07	31,0	5,1	-19,8	2,40	2,42	2,43
74,47	32,6	6,4	-19,8	12,95	11,50	12,17
84,62	-21,6	33,3	70,0	2,91	2,99	3,46
87,97	114,3	170,8	26,4	2,79	3,02	3,40
48,66	47,7	6,7	-27,8	2,79	2,97	2,88
26,33	-91,9	-70,7	262,2	2,77	3,90	3,32

**PerChYKL40_2_1**	**PerChYKL40_3_1**	**PerChYKL40_3_2**	PIINP_1	PIINP_2	PIINP_3
7,7	0,3	-6,9	9,55	10,97	12,62
-6,3	-6,8	-0,5	11,91	20,07	16,77
-6,6	-11,0	-4,7	10,27	10,47	10,81
32,6	32,6	0,0	5,29	6,06	5,03
-10,2	66,3	85,2	7,78	7,22	9,58
5,9	-1,4	-6,9	9,50	12,49	13,58
-2,4	-9,0	-6,7	6,52	12,08	11,67
-9,6	-23,0	-14,8	11,32	9,76	11,33
10,9	13,5	2,4	6,91	6,80	6,78
2,5	3,8	1,2	8,95	13,34	10,11
-0,7	-11,8	-11,2	8,88	11,98	11,58
0,8	1,3	0,4	13,61	15,13	16,55
-11,2	-6,0	5,8	7,79	12,53	9,78
2,7	18,9	15,7	9,16	9,06	9,80
8,2	21,9	12,6	10,72	8,57	12,18
6,5	3,2	-3,0	9,16	8,66	9,19
40,8	19,9	-14,9	6,46	8,69	9,61

PerChPIINP2_1	PerChPIINP3_1	PerChPIINP3_2	PIIANP_1	PIIANP_2	PIIANP_3	PerChPIIANP_2_1
14,9	32,1	15,0	2164,40	1806,24	2018,83	-16,5
68,5	40,8	-16,4	1797,35	1167,55	1646,27	-35,0
1,9	5,3	3,2	1745,21	1548,73	1681,27	-11,3
14,6	-4,9	-17,0	1928,06	1811,22	1299,64	-6,1
-7,2	23,1	32,7	2852,78	2014,07	1763,37	-29,4
31,5	42,9	8,7	2446,40	1788,82	2699,22	-26,9
85,3	79,0	-3,4	3230,49	3694,45	2181,21	14,4
-13,8	0,1	16,1	2021,97	1986,94	1734,59	-1,7
-1,6	-1,9	-0,3	1662,52	1836,81	1629,53	10,5
49,1	13,0	-24,2	1948,51	1651,35	1870,45	-15,3
34,9	30,4	-3,3	1562,64	1191,55	1273,76	-23,7
11,2	21,6	9,4	3128,29	1937,94	1793,49	-38,1
60,8	25,5	-21,9	1751,87	1690,06	1343,43	-3,5
-1,1	7,0	8,2	1360,03	1466,65	1260,27	7,8
-20,1	13,6	42,1	1369,86	1536,53	1417,15	12,2
-5,5	0,3	6,1	1629,86	1736,09	2553,12	6,5
34,5	48,8	10,6	2114,41	2073,91	1916,91	-1,9

PerChPIIANP_3_1	PerChPIIANP_3_2	C2C_1	C2C_2	C2C_3	PerChC2C_2_1
-6,7	11,8	242,72	201,72	245,19	-16,9
-8,4	41,0	236,61	152,38	262,20	-35,6
-3,7	8,6	229,16	183,84	194,53	-19,8
-32,6	-28,2	247,82	188,82	186,53	-23,8
-38,2	-12,4	248,73	188,57	254,26	-24,2
10,3	50,9	248,62	242,75	260,60	-2,4
-32,5	-41,0	248,87	239,09	273,70	-3,9
-14,2	-12,7	282,23	210,43	308,22	-25,4
-2,0	-11,3	253,63	231,45	283,13	-8,7
-4,0	13,3	252,46	226,19	217,69	-10,4
-18,5	6,9	207,01	205,40	211,93	-0,8
-42,7	-7,5	164,17	99,11	163,39	-39,6
-23,3	-20,5	171,4	166,64	160,66	-2,8
-7,3	-14,1	183,09	151,39	180,14	-17,3
3,5	-7,8	199,8	142,71	176,52	-28,6
56,6	47,1	136,15	168,96	185,22	24,1
-9,3	-7,6	201,62	207,94	204,66	3,1

PerChC2C_3_1	PerChC2C_3_2
1,0	21,5
10,8	72,1
-15,1	5,8
-24,7	-1,2
2,2	34,8
4,8	7,4
10,0	14,5
9,2	46,5
11,6	22,3
-13,8	-3,8
2,4	3,2
-0,5	64,9
-6,3	-3,6
-1,6	19,0
-11,7	23,7
36,0	9,6
1,5	-1,6
